# Supplementary figures and images for: Marine Litter Tracking System: A Case Study with Open-Source Technology and a Citizen Science-Based Approach
Source: Sensors (Basel). 2023 Jan 13;23(2):935. doi: 10.3390/s23020935 (PMC9863889; doi:10.3390/s23020935)

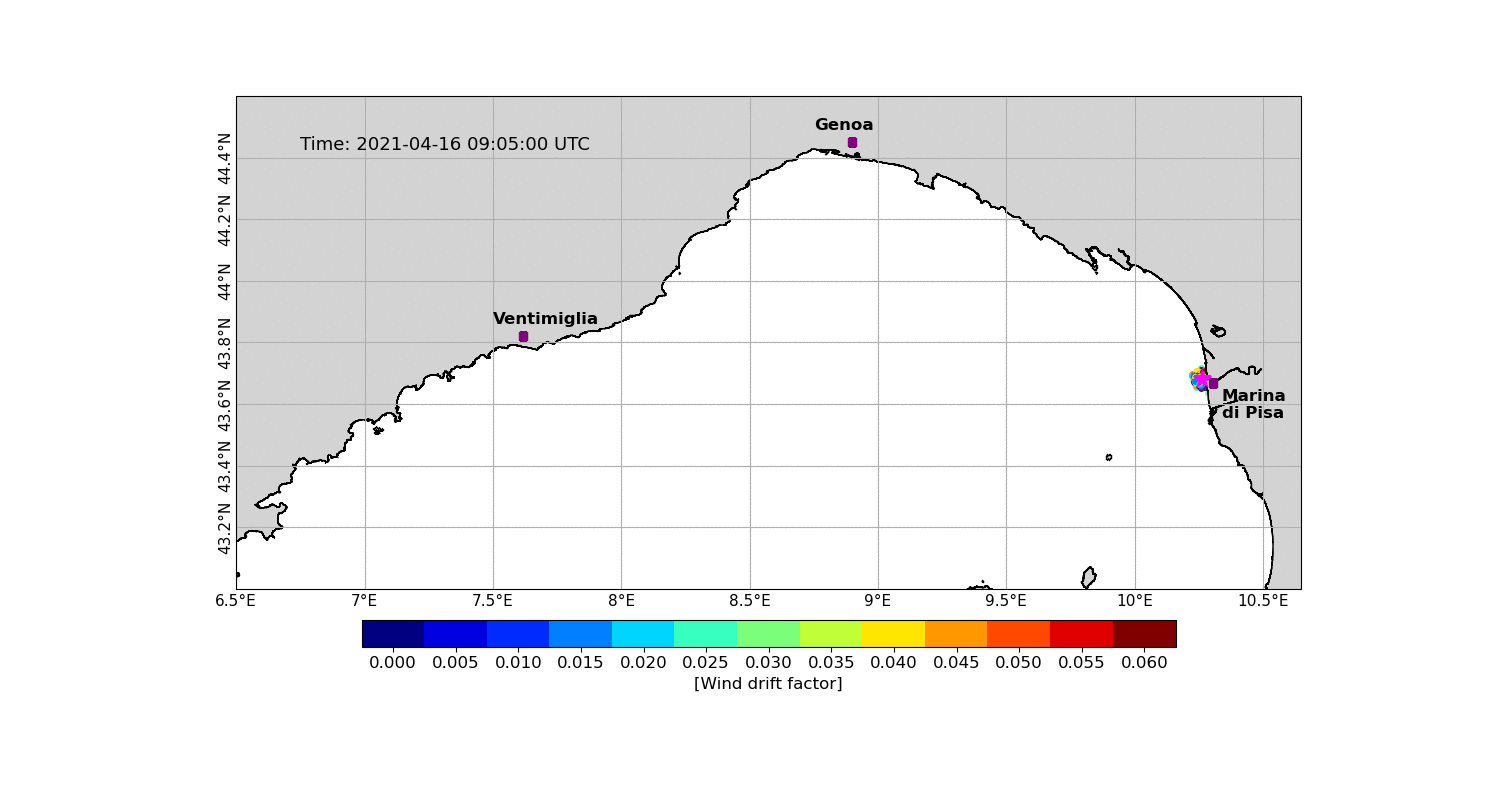

Supplement: Supplementary file 1 [file sensors-23-00935-s001.zip › GIF_FILES/20210416_wind_current_L1T1.gif]

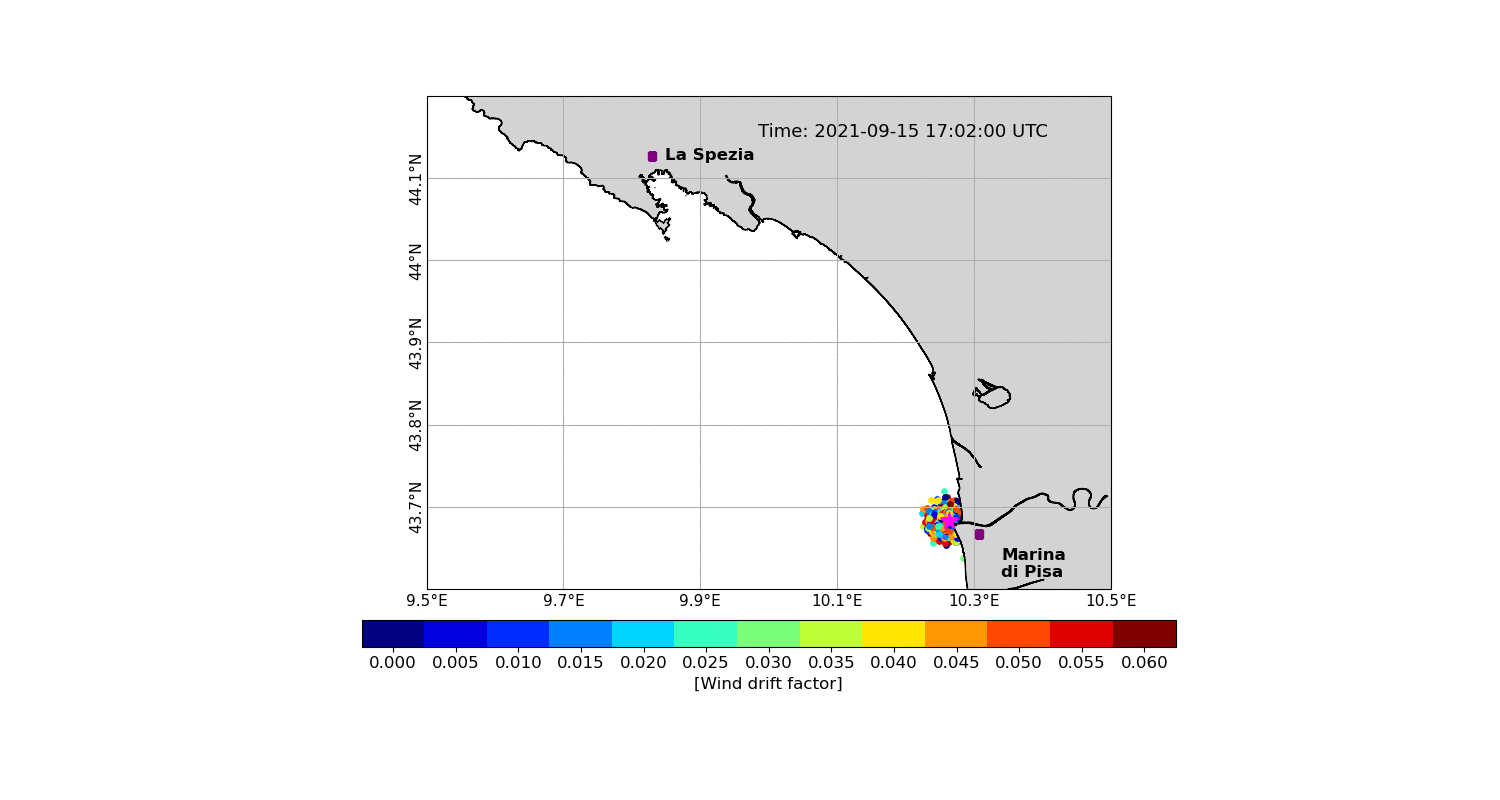

Supplement: Supplementary file 1 [file sensors-23-00935-s001.zip › GIF_FILES/20210915_wind_current_L2T2.gif]

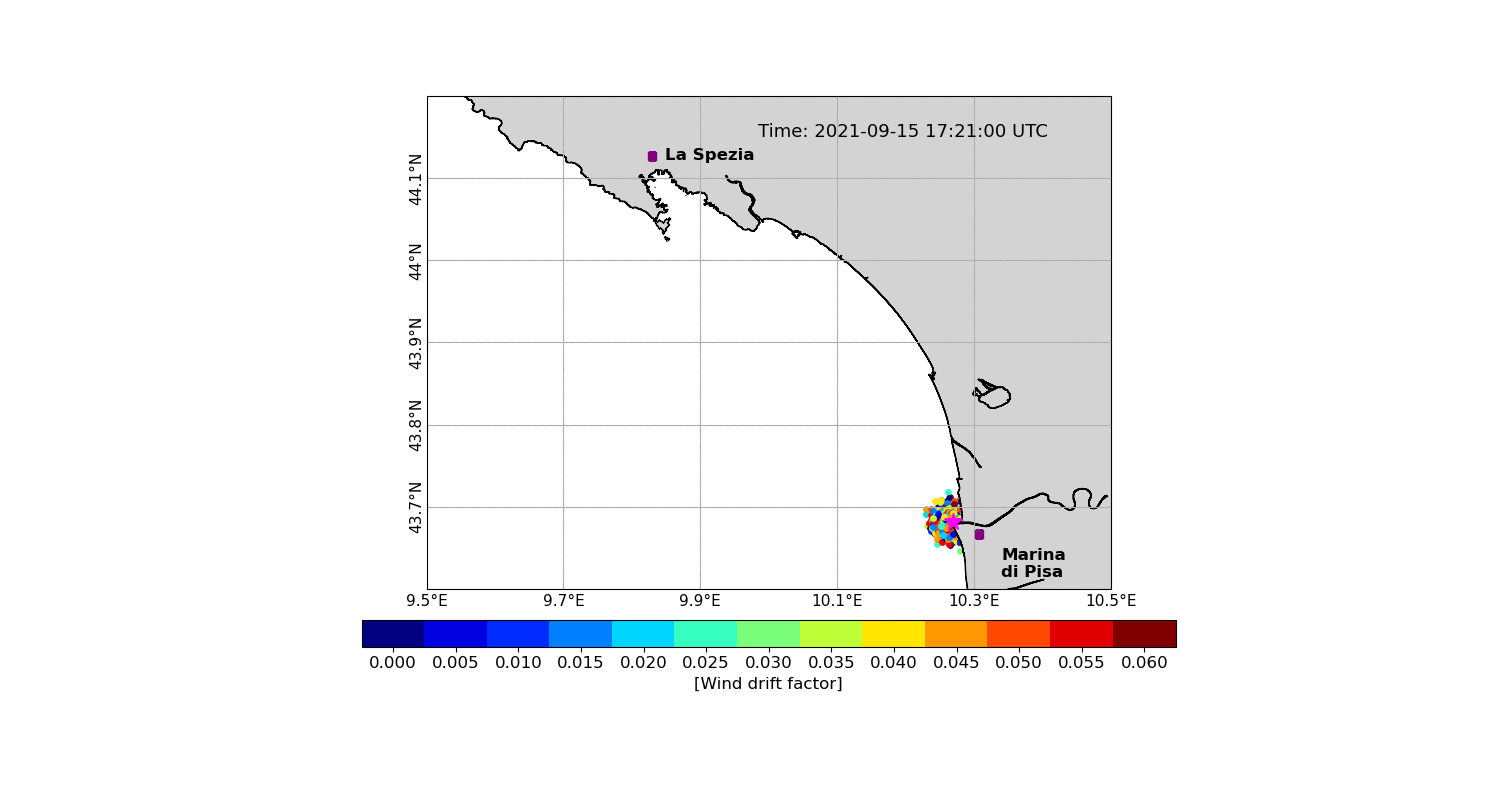

Supplement: Supplementary file 1 [file sensors-23-00935-s001.zip › GIF_FILES/20210915_wind_current_L3T1.gif]

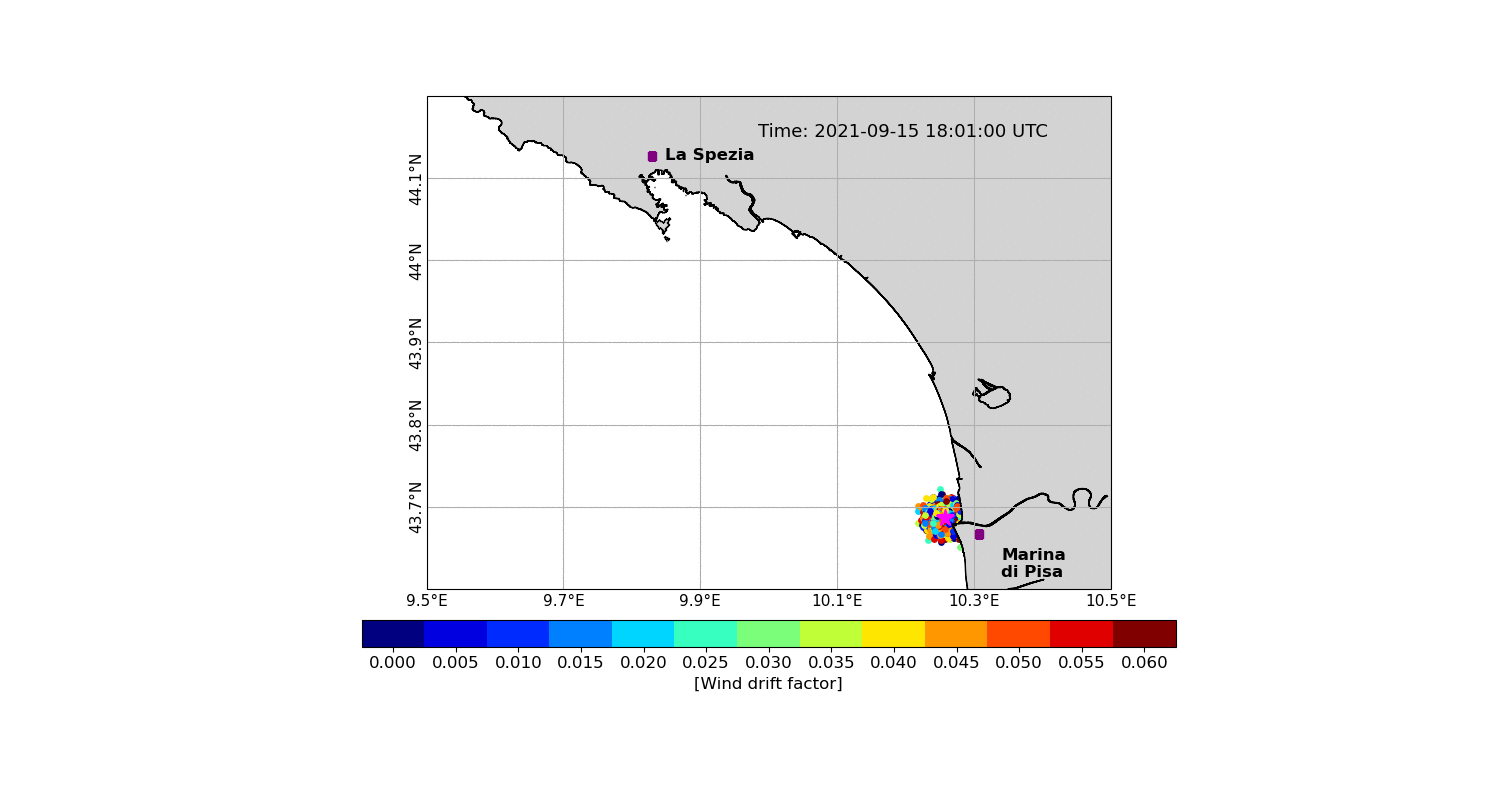

Supplement: Supplementary file 1 [file sensors-23-00935-s001.zip › GIF_FILES/20210915_wind_current_L3T2.gif]

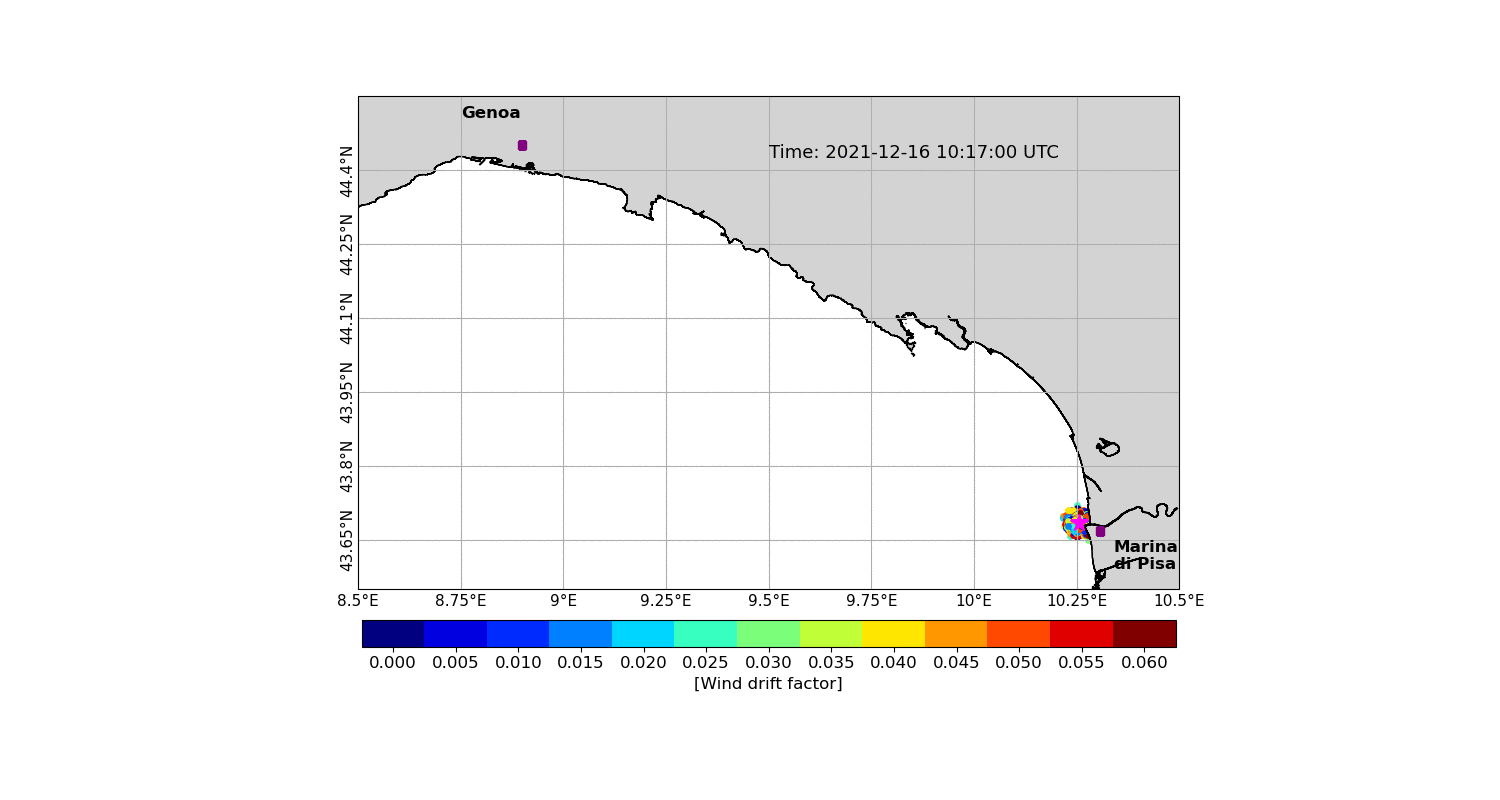

Supplement: Supplementary file 1 [file sensors-23-00935-s001.zip › GIF_FILES/20211216_wind_current_L2T1.gif]

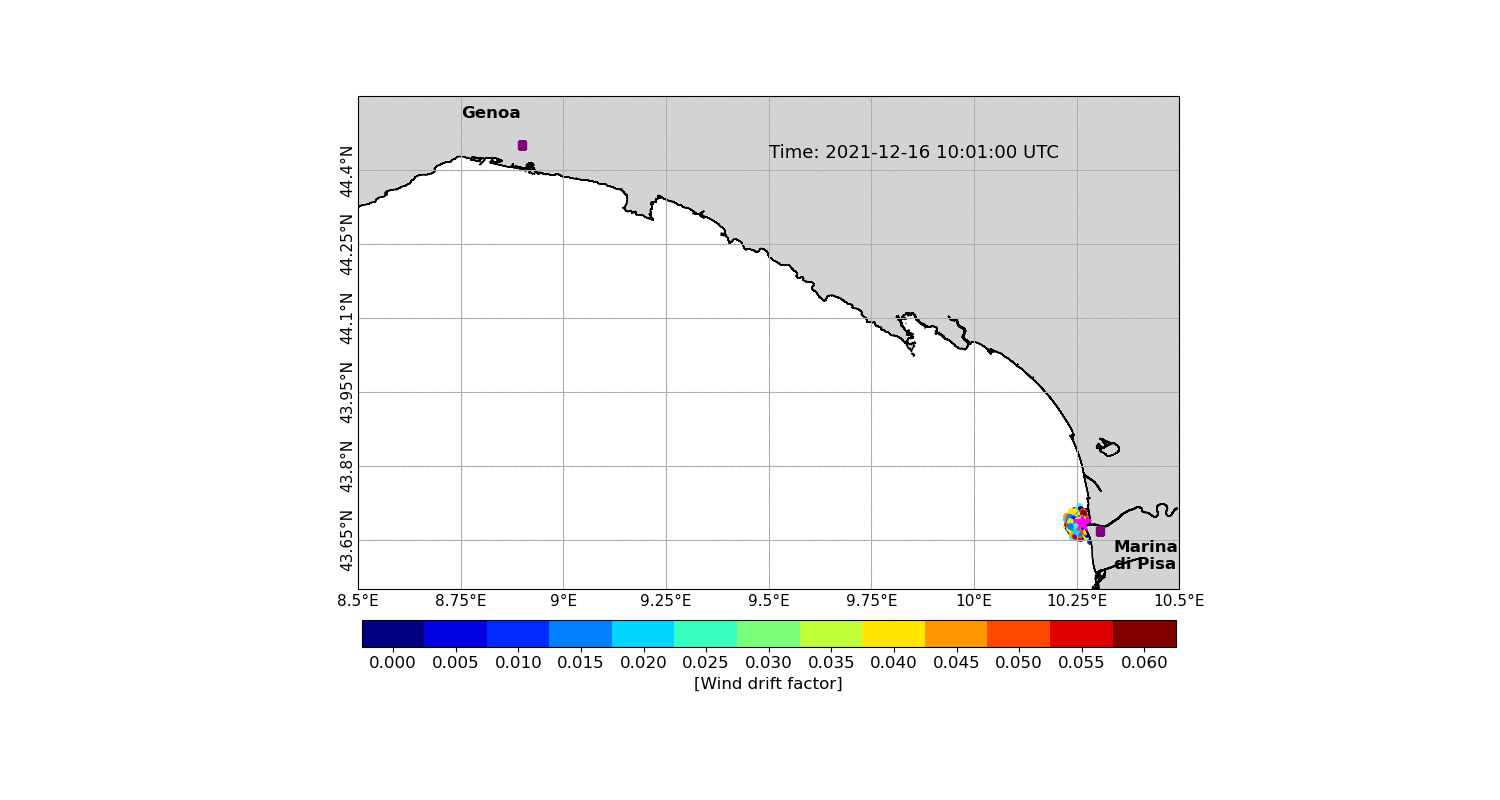

Supplement: Supplementary file 1 [file sensors-23-00935-s001.zip › GIF_FILES/20211216_wind_current_L3T1.gif]

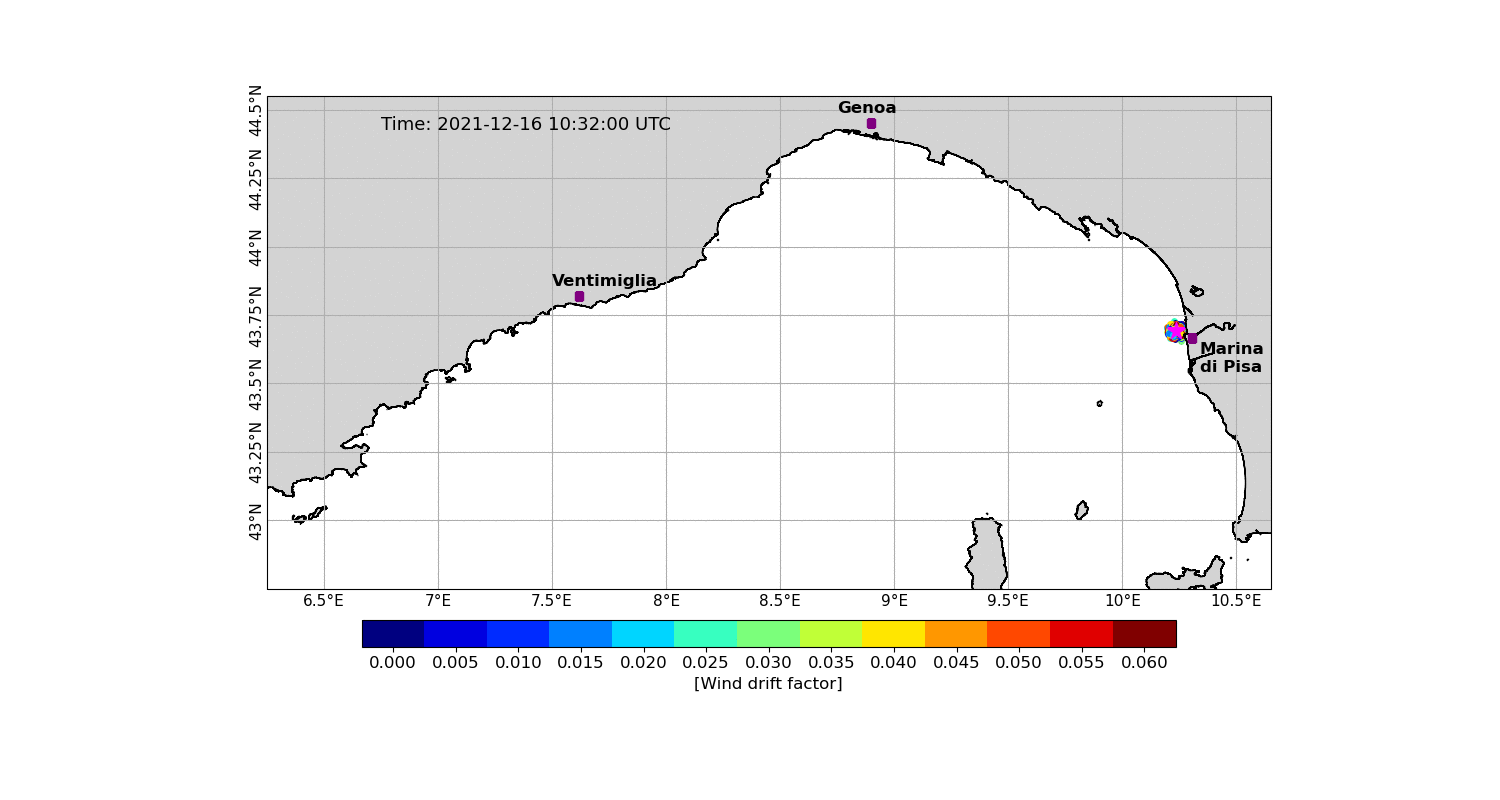

Supplement: Supplementary file 1 [file sensors-23-00935-s001.zip › GIF_FILES/20211216_wind_current_L4T1.gif]

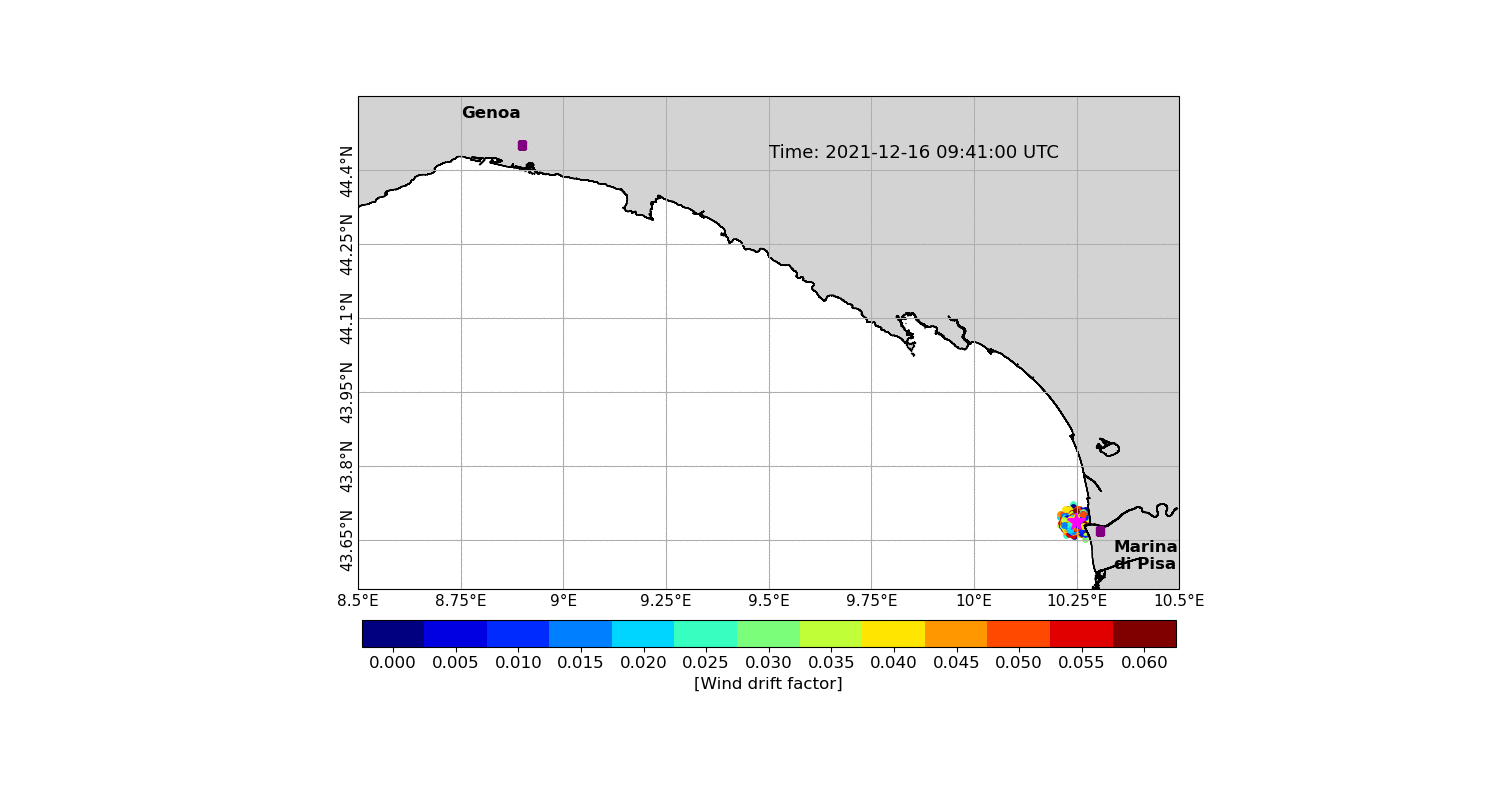

Supplement: Supplementary file 1 [file sensors-23-00935-s001.zip › GIF_FILES/20211216_wind_current_L4T3.gif]
